# Arthroscopic evaluation for omalgia patients undergoing the clavicular hook plate fixation of distal clavicle fractures

**DOI:** 10.1186/1749-799X-9-46

**Published:** 2014-06-11

**Authors:** Xin Gu, Biao Cheng, Jian Sun, Kun Tao

**Affiliations:** 1 Department of Orthopaedic, Shanghai Tenth People's Hospital, Tongji University, No. 301 Middle Yanchang Road, Zhabei District, Shanghai 20072, China

**Keywords:** Clavicular hook plate, Distal clavicle fractures, Shoulder arthroscopy

## Abstract

**Background:**

The aim of this study is to investigate the anatomic changes in the shoulder joints responsible for omalgia after the clavicular hook plate fixation under arthroscope.

**Methods:**

Arthroscopic examination was carried out for 12 omalgia patients who underwent clavicular hook plate fixation due to distal clavicle fractures. Functional outcome of shoulder was measured by the Japanese Orthopaedic Association (JOA) score before and after the withdrawal of the fixation plate.

**Results:**

The rotator cuff compression by the clavicular hook was arthroscopically observed in 11 of the 12 cases. The JOA scores of the shoulder were significantly improved at 1 month after the withdrawal of the fixation plate (pain, 28 ± 2.4 vs. 15 ± 5.2; function, 19.2 ± 1.0 vs. 11.7 ± 1.9; range of movements, 26.8 ± 2.6 vs. 14.8 ± 3.4) compared with before.

**Conclusions:**

The impingement of the hook to the rotator cuff may be the main cause for the omalgia. The appropriate hook and plate that fit to the curve of the clavicle as well as the acromion are necessary to decrease the severity of omalgia.

## Introduction

Distal clavicle fractures account for approximately 10%–26% of all the clavicle fractures, which can be mainly caused by car accidents and sports injuries [1]. Neer further divides the distal clavicle fractures into three types according to the relation of the fracture line to the coracoclavicular ligaments, among which type II fractures occur medially (IIA) or laterally (IIB) to the coracoclavicular ligaments and often result in major displacement because of complete or incomplete rupture of the coracoclavicular ligaments [2]. Although non-surgical strategies can be effective for the treatment of type II distal clavicle fractures, they lead to higher non-union rates (> 30%) [3-5]. Therefore, a series of surgical techniques have been developed in order to reduce the non-union rate and improve functional outcome, including clavicular hook plate [6-8]. Recent studies suggest that the use of a clavicular hook plate is a reasonable option for fixation of unstable distal clavicle fractures, providing a high rate of union and satisfactory functional results [9,10]. However, several patients complain of postoperative omalgia [11] which may be relieved after removal of the hook plate [12].

Recently, it is hypothesized that the omalgia after the clavicular hook plate fixation results from the mechanical hook impingement on the subacromial shoulder and rotator cuff based on radiography [13], magnetic resonance imaging (MRI) [14,15], and sonography [11,16]. In this study, we aimed to further evaluate the anatomic changes in 12 patients who underwent AO clavicular hook plate fixation for distal clavicle fractures and suffered from postoperative omalgia using arthroscopy. Compared with radiography, MRI, and sonography, arthroscopy is not only effective to diagnose but minimally invasive to visualize and treat the shoulder problems.

## Patients and methods

Our study was conducted on 12 patients who suffered from trauma-induced distal clavicle pain and limited movements and then were diagnosed in our hospital as distal clavicle fractures (Neer II) (seven males and five females, average age 46.1 years, range 31–68 years). Open reduction and clavicular hook plate (AO, Synthes, Bettlach, Switzerland) internal fixation was performed within 2 weeks (mean time, 3.1 days) after the injuries. Patients received moderate suspension fixation for 2 weeks and then functional exercise. Postoperatively, all patients complained of omalgia, limited shoulder movements, or friction. After being followed up until fractures healed, the patients were requested to undergo arthroscopy to check clavicle hook position and status of rotator cuff and then received removal of hook plate (Synthes, Oberdorf, Switzerland). The patients were further followed up to observe the recovery of shoulder function after the removal of the implant. This study was conducted with the approval of the ethics committee of Shanghai Tenth People's Hospital, and all patients gave written informed consent.

Our surgical procedure was performed with the patient in the beach chair position after general anesthesia and routine disinfection. A 3.5-mm 30° arthroscope (Stryker, Warsaw, IN, USA) was placed through the posterior and anterior portals of the shoulder joint to observe the coracoclavicular ligaments, distal clavicle, acromioclavicular joints, acromion, subacromial clavicular hook, and rotator cuff. The contact and compression between the clavicular hook and rotator cuff were also recorded under the safe range of movements. Adhesiolysis was performed for the subacromial soft tissue adhesion after the removal of the plate. Ice compression was applied postoperatively, passive functional exercise was allowed 1 day postoperatively and for 3 weeks, and then active functional exercise permitted. Physiotherapy was given during the whole process.

The Japanese Orthopaedic Association (JOA) scoring system was used to evaluate the shoulder joint function [17], and the scores were taken before and at 1 month after the removal of plate. The JOA scoring system includes shoulder pain assessment (30 points), shoulder function (20 points), range of movement (ROM) (30 points), radiographic evaluation (5 points), and shoulder stability (15 points). The pain, shoulder function, and ROM scores were compared before and after the removal of plate using the paired *t* test with the SPSS 10.0 software (SPSS, Chicago, IL, USA). *P* < 0.05 was considered statistically significant.

## Results

The 12 patients had suffered from shoulder pain and limited movement since distal clavicle was fractured and after the plate internal fixation. The scores for shoulder pain, shoulder function, and ROM before the removal of plate were 15 ± 5.2, 11.7 ± 1.9, and 14.8 ± 3.4, which were significantly improved at 1 month after the removal of plate (shoulder pain, 28 ± 2.4; shoulder function, 19.2 ± 1.0; ROM, 26.8 ± 2.6) (Table 1). Ten patients were able to do slight physical activities.

**Table 1:** Average JOA score of the shoulder before and 1 month after removal of fixation plate

	**JOA score of shoulder joint**				
	**Pain**	**Function**	**ROMs (elevating + external rotation + internal rotation)**	**Radiographic evaluation**	**Stability**
Preoperative	15 ± 5.2	11.7 ± 1.9	14.8 ± 3.4	5	15
Postoperative	28 ± 2.4	19.2 ± 1.0	26.8 ± 2.6	5	15
*t*	11.8	12.1	10.5		
*p*	< 0.001	< 0.001	< 0.001		

*ROM* range of movement, *JOA* the Japanese Orthopaedic Association.

**Figure 1:**
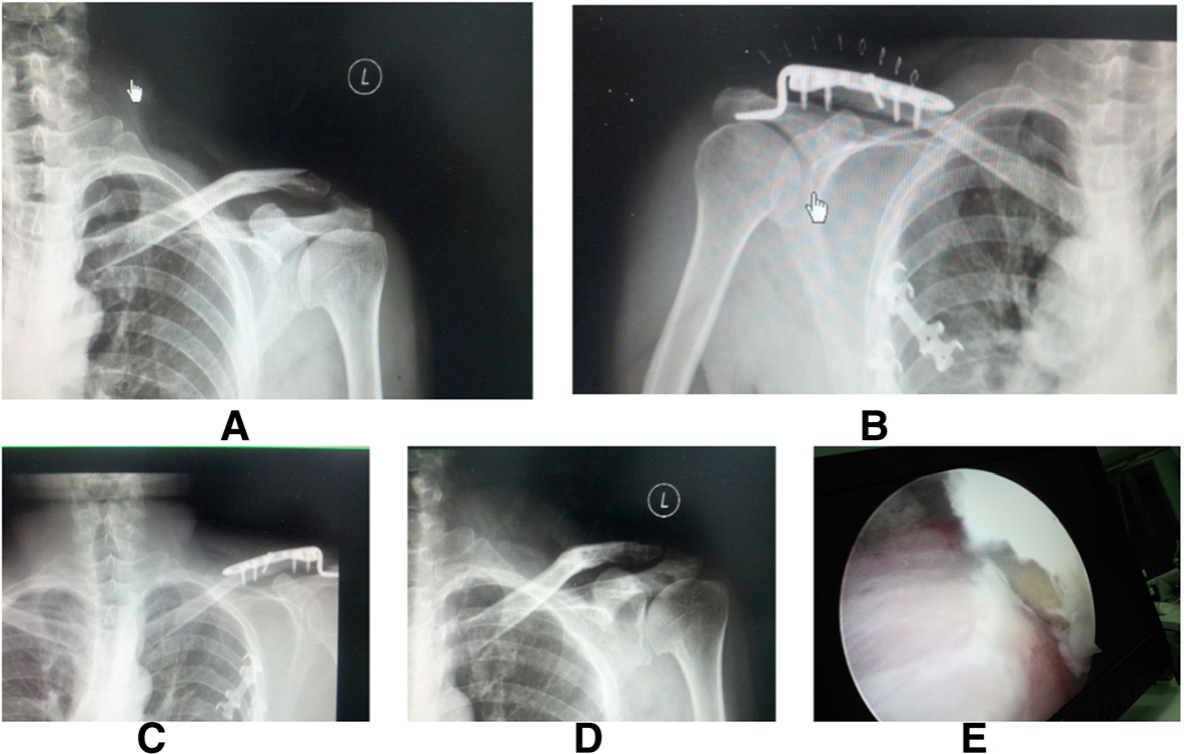
**Injury and preoperative and postoperative status of a 56-year-old man.** Left distal clavicle fracture (Neer type II) **(A)**. Hook plate fixation **(B)**. The bone was healed 1 month after surgery, but the patient still complained of omalgia **(C)**. After removal of internal fixation **(D)**, extrusion of clavicular hook on supraspinatus was observed **(E)** under shoulder arthroscopy.

**Figure 2:**
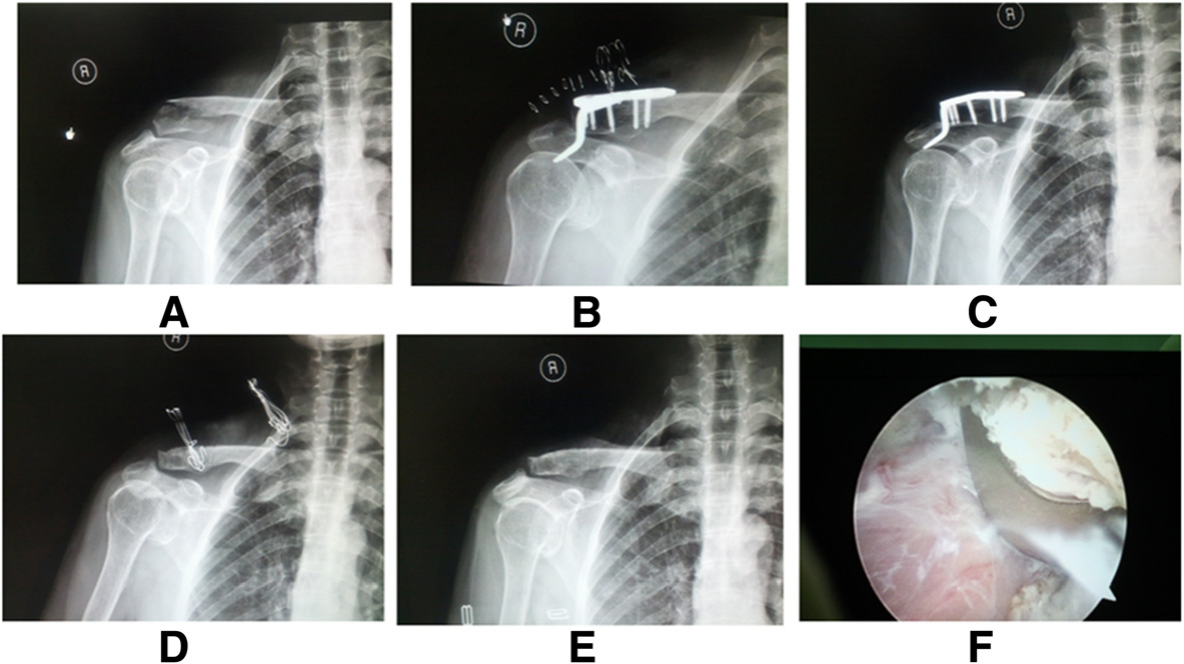
**Injury and preoperative and postoperative status of a 68-year-old woman.** Right distal clavicle fracture (Neer type II) **(A)**. Clavicular hook plate fixation was performed, but the hook plate was not modulated well **(B)**. Patient complained of omalgia after internal fixation for 3 months **(C)**. After removal of internal fixation **(D)** and 1 month later **(E)**, omalgia was relieved. The arthroscopy revealed that the hook plate compressed the supraspinatus muscle **(F)**.

Arthroscopy indicated that except one patient manifested subacromial osteophytosis-induced impingement on the acromion; other patients exhibited compression of the clavicular hook on the supraspinatus, obstructing the external rotation of the shoulder. The proximal clavicular hook was the main part compressing the supraspinatus tendon. Even, in one patient whose posterior tip of the clavicle hook went downwards, the whole clavicle hook contacted with the rotator cuff. In another case, tear notch was observed in the rotator cuff against the compression of the clavicle hook. The general information for the 12 patients is shown in Table 2, and a typical case is shown in Figures 1 and 2.

**Table 2:** Details of the 12 patients

**Number**	**Gender/age**	**Plate (number of holes, height (mm))**	**Weeks of healing**	**Preoperative JOA scores (scores for radiographic evaluation and stability were not shown)**			**Arthroscopy**	**Postoperative JOA scores (scores for radiographic evaluation and stability were not shown)**		
				**Pain**	**Function**	**ROM (elevating + external rotation + internal rotation)**		**Pain**	**Function**	**ROM (elevating + external rotation + internal rotation)**
1	M/31	6, 15	12	20	12	9 + 1 + 2	Rotator cuff involvement by proximal hook, subacromial osteophytes	30	20	12 + 6 + 6
2	F/56	8, 18	11	20	11	6 + 3 + 2	Rotator cuff involvement by proximal hook	30	20	12 + 9 + 6
3	M/49	6, 18	13	10	13	9 + 3 + 4	Rotator cuff involvement by proximal hook	30	18	12 + 6 + 6
4	M/62	6, 18	10	10	9	6 + 1 + 2	Rotator cuff involvement by the whole hook	25	18	12 + 6 + 6
5	F/37	6, 15	14	20	14	9 + 3 + 4	Subacromial osteophytes, scar adhesions	30	17	15 + 9 + 6
6	F/42	8, 18	12	10	12	6 + 2 + 2	Rotator cuff involvement by proximal hook, partial injury	30	20	15 + 9 + 6
7	M/38	6, 18	11	10	11	9 + 3 + 4	Rotator cuff involvement by proximal hook	25	20	15 + 9 + 4
8	M/40	6, 18	12	10	9	9 + 6 + 2	Rotator cuff involvement by proximal hook	25	19	15 + 6 + 4
9	M/33	6, 18	12	20	12	9 + 3 + 4	Rotator cuff involvement by proximal hook	30	19	15 + 9 + 6
10	F/38	6, 15	14	20	14	9 + 6 + 4	Rotator cuff involvement by proximal hook	30	20	15 + 6 + 4
11	M/59	6, 18	9	10	9	9 + 6 + 4	Rotator cuff involvement by proximal hook	25	19	15 + 9 + 6
12	F/68	4, 15	14	20	14	9 + 3 + 4	Rotator cuff involvement by proximal hook	30	20	15 + 6 + 4

*ROM* range of movement, *JOA* the Japanese Orthopaedic Association, *M* male, *F* female.

## Discussion

According to the Neer classification, in the type II distal clavicle fractures, the proximal fragment is very unstable and has no ligamentous attachment, and the muscle forces and weight of the arm tend to displace the fracture fragments, which are difficult to be reset by non-operative management and cause the high rate of non-union compared with other types [2]. Therefore, a number of surgical procedures have been developed to reduce the occurrence of non-union and the morbidity period, such as fixations via Kirschner wires and tension band on the acromion and acromioclavicular joint [18] and fixation between the clavicle and coracoid using screw [19], suture anchor, and plate [20-23]. Among them, the clavicular hook plate fixation is the most widely adopted and reported approach [9,11,24].

The AO plate introduced in 1997 in Europe [25] and in 2002 in Asia is the most widely used clavicular hook plate [11-13,23,26,27] (Table 3). The clavicle hook is inserted below the acromion posterior to the acromioclavicular joint and resets the distal clavicle by the lever principle to disperse the stress of fracture displacement to the acromion, thus providing a good stability. It is reported that there is a small rotation between the scapula and clavicle during abduction and flexion of the upper arm [28]. The rigid fixation of the acromioclavicular joint may lead to a shifting and loosening of the internal fixation. Clavicular hook plate is designed to reduce the interference with the acromioclavicular joint but allow micromotion [28,29]. However, clavicular hook placed under the acromion also can lead to various complications: Rotator cuff and shoulder joint capsule would be impinged if the clavicle hook is placed deeply or the subacromial space is small. Subacromial osteolysis and even unhooking take place if the subacromial stress is too concentrated. Among them, acromial impingement is the most common complication. Renger and others indicate that the incidence of shoulder joint friction is as high as 68%, and shoulders cannot reach approximately 90° of abduction [1]. In the present study, omalgia was also reported in all of the 12 patients who mainly complained of limited movements in lifting and joint friction. Arthroscopy indicated that rotator cuff supraspinatus was compressed in 11 of the 12 patients, and the main part for application of a force was the proximal clavicular hook, which was in accordance with the study described by ElMaraghy et al. [30].

**Table 3:** AO hook plate for the treatment of the Neer type II distal clavicle fractures

**Author**	**Year**	**Country**	**Case number (M/F)**	**Type of hook plate**	**Hardware removal (time after fixation operation,** ***N*)**	**Impingement rate**	**Impingement diagnosis method**
Flinkkilä et al. [26]	2002	Finland	17 (16/1)	AO plate (Stratec Medical, Oberdorf, Switzerland)	5 months, 17	**-**	**-**
Muramatsu et al. [13]	2007	Japan	15 (13/2)	Synthes AO plate	4.5 months, 12	0%	Radiography
Lee et al. [27,34]	2009	Taiwan	32 (14/18)	Synthes AO plate	4.8 months, 32	0%	Radiography
Hsu et al. [23]	2010	Taiwan	35 (23/12)	AO plate (Synthes, West Chester, PA, USA)	1 year, 35	25.7% (9/35)	Radiography
Leu et al. [11]	2012	Taiwan	25 (13/12)	AO plate (Synthes, Bettlach, Switzerland)	5.8 months, 25	36% (9/25)	Sonography
Tan et al. [12]	2012	China	23 (15/8)	AO plate	3–14 months, 15	–	–

*M* male, *F* female, *N* number of patients who underwent the hardware removal.

To reduce the interference with the acromioclavicular joint, the AO hook plate should be designed to fit anatomically to the acromion and clavicle [31]. In this study, we observed that the whole clavicular hook was imposed on the supraspinatus due to the downward bending of the hook in one case, which led to soft tissue adhesion and small ROM. Smaller hook has been suggested by some researchers to reduce subacromial influence, but it increases the pressure on acromion as well as the risks of acromial osteolysis, unhooking, and stress fractures. By now, there are no standard data for the length, width, thickness, and coracoclavicular distance [30,32,33] because of the individual variance. There are only two types of plate according to the height of the hook (15 or 18 mm), which do not match completely with the different individuals and contributes to the high incidence of complications.

Shoulder arthroscopic examination can be an effective tool to detect rotator cuff tissue injury and thus helps to recover the shoulder function. Rotator cuff compression was observed in 11 of the 12 patients in the present study, and partial tear of the rotator cuff was seen in 1 case. In cadaveric specimens, ElMaraghy et al. find that clavicle hook can pierce the subacromial bursa and induce bursitis, which ultimately results in impingement [30]. Since arthroscopic examination was conducted after the fractures were healed, subacromial bursitis disease could not be investigated, but soft tissue adhesion was detected in our study. Recently, arthroscopy is also introduced to clavicular plate fixation surgery to adjust the plate location and avoid impingement complication. For example, Lee et al. used the arthroscopic-assisted locking compression plate fixation for the treatment of unstable fractures of the lateral end of the clavicle, and only 1 of 23 patients reported shoulder pain due to bursitis [34]. Gille et al. combined arthroscopy and hook plate fixation to treat acromioclavicular joint dislocation and decrease risks related to open surgery [35]. Nourissat et al. raise an arthroscopic-assisted surgical procedure to stabilize the fracture and reconstruct the ligament, which allows for total recovery of shoulder function without inconvenience from device [36]. Takase and others also develop a similar surgical solution [37].

Moreover, rapid improvements in the shoulder function were observed for all the patients after the removal of the plate. It proved that subacromial interference of clavicular hook plate was the main source of postoperative omalgia. Nowadays, most researchers believe that moderate activity restriction is necessary after clavicular hook plate fixation surgery and removal of the plate should be conducted as soon as the fracture is healed [1,38,39].

However, there are some limitations in this study. Firstly, this study is a retrospective case series, and the evidence level of which is lower than that of a cohort study and prospective study. Secondly, because Neer type II distal clavicle fractures are less common, it was difficult to obtain a sufficient number of patients. Small sample size leads to only one case showing the rotator cuff tear. Thus, the classifications and etiopathogenesis of rotator cuff tears were not investigated [15,40,41]. Thirdly, the follow-up period was short for the evaluation of shoulder joint function after the removal of plate. Therefore, future studies need to be performed with longer-term monitoring and larger study populations from multiple medical centers to verify our results.

## Conclusion

Overall, our study investigated the detailed impact of clavicular hook plate on acromion with arthroscopy, which provided information for future improvements to reduce the incidence of complications, especially omalgia. Applying a plate completely fitting the acromion and clavicle minimizes subacromial interference and the stress on acromion. Since preoperative X-ray cannot show the three-dimensional structure of the acromioclavicular joint, digital construction of the structure and development of individualized clavicular hook plate in terms of shape and location are the directions of future researches.

## Competing interests

The authors declare that they have no competing interests.

## Authors' contributions

XG and BC participated in the design of this study, and they both performed the statistical analysis. JS carried out the study, together with KT, collected important background information, and drafted the manuscript. BC conceived of this study, participated in the design, and helped draft the manuscript. All authors read and approved the final manuscript.
